# Fluazuron Baits in the Control of *Amblyomma sculptum* Tick: Efficacy and Pharmacokinetics Using Guinea Pigs as an Experimental Model

**DOI:** 10.3390/pathogens14090854

**Published:** 2025-08-28

**Authors:** Debora Azevedo Borges, Isabelle Vilela Bonfim, Clara Rodrigues Dutra, Ingrid Lins Raquel de Jesus, Rayane Monteiro, Rayane Christine Pereira de Assis, Fernando Rocha Miranda, Yara Peluso Cid, Fabio Barbour Scott

**Affiliations:** 1 Veterinary Science, Veterinary Institute, Federal Rural University of Rio de Janeiro, Seropédica 23897-000, Brazil; isabelle_vilela@hotmail.com (I.V.B.); clararodriguesdutra@gmail.com (C.R.D.); raquellingrid@gmail.com (I.L.R.d.J.); rayanecpassis@gmail.com (R.C.P.d.A.); fernando_miranda02@hotmail.com (F.R.M.); 2 Veterinary Medicine, Veterinary Institute, Federal Rural University of Rio de Janeiro, Seropédica 23897-000, Brazil; monteirorayane918@gmail.com; 3 Pharmaceutical Science Department, Health and Biological Science Institute, Federal Rural University of Rio de Janeiro, Seropédica 23897-000, Brazil; yaracid@ufrrj.br; 4 Animal Parasitology Department, Veterinary Institute, Federal Rural University of Rio de Janeiro, Seropédica 23897-000, Brazil; scott.fabio@gmail.com

**Keywords:** tick star, benzoylphenyl ureas, tick control

## Abstract

The tick *Amblyomma sculptum* is the vector of the bacterium *Rickettsia rickettsii* (which causes Brazilian Spotted Fever in humans). It can be found in domestic and wild animals, as well as humans. The objective of this work was to evaluate the efficacy of baits containing fluazuron in the control of *A. sculptum* in guinea pigs that were artificially infested. The work was conducted in two studies: descriptive pharmacokinetics and efficacy. Descriptive pharmacokinetics was assessed after administering one fluazuron-containing bait per animal (10 mg/kg). To determine the pharmacokinetic profile, blood samples were collected over several days. For the efficacy test, the animals were divided into four groups: a control group (untreated) and three treated groups that received a single dose of 10 mg/kg on days −21 (G1), −14 (G2) and −7 (G3). All animals were infested with *A. sculptum* larvae on day 0 and recovered on days +4, +5, +6, and +7. Fluazuron plasma concentrations increased rapidly, indicating rapid absorption, and decreased slowly. In all treated groups, engorged larvae with morphological and behavioral changes were observed. Although fluazuron did not show acaricidal efficacy, it was able to interfere with tick molting. Fluazuron was effective in controlling the *A. sculptum* tick in guinea pigs.

## 1. Introduction

The tick *Amblyomma sculptum* is an ixodid belonging to the *Amblyomma cajennense* Complex. Any of its parasitic phases can be found in domestic and wild animals, and even in humans [1]; however, their preferred hosts are tapirs, horses, and capybaras [2,3]. Its bite causes extremely itchy lesions. It is the species that transmits the bacterium *Rickettsia rickettsii*, which causes Brazilian Spotted Fever (BSF) in humans [4]. This species can be found in several areas of South America [1].

Capybaras are considered the most problematic hosts of *A. sculptum*, mainly because they can serve as amplifier hosts of the bacterium *R. rickettsii* [5] and due to their high population density in both natural and anthropic environments [6], necessitating tick control.

Some field studies have been conducted to control ectoparasites in rodents using wildlife-targeted delivery systems, most of which employed benzoylphenyl ureas (e.g., fluazuron, lufenuron, pyriproxyfen), all with the shared goal of reducing populations of ectoparasites that serve as vectors of microorganisms causing lethal diseases in humans [7,8,9]. Furthermore, fluazuron (10 mg/kg) has demonstrated efficacy in controlling *A. sculptum* in guinea pigs artificially infested with larvae when administered via gavage [9].

Therefore, given the need to develop methods of administering acaricides to control *A. sculptum* in wild rodents, including capybaras, due to their public health relevance, this study aimed to evaluate the effectiveness of baits containing fluazuron in controlling *A. sculptum* using guinea pigs as an experimental model, as part of the initial exploration of molecules with acaricidal potential for use in wild rodents.

## 2. Materials and Methods

The work was conducted in two studies approved by the Ethics Committee of the Veterinary Institute of the Federal Rural University of Rio de Janeiro: descriptive pharmacokinetics (code: 7690060821) and efficacy (code: 6648090222).

All guinea pigs included in the studies were clinically healthy, approximately 6 months old, weighing ±500 g, untreated with ectoparasiticides, and housed individually in cages (0.60 m height × 1.2 m width × 0.60 m depth) placed on a masonry floor. They had ad libitum freshwater supply and were fed dry food twice daily according to each animal’s need. The animals were kept in a temperature-controlled room at 21 ± 1 °C using air conditioning. Environmental enrichment measures were adopted to reduce stress caused by the confinement and management necessary to conduct the studies.

The developed baits were adapted from the publications of [7,8]. The animals of the treated groups received baits containing 5 mg fluazuron each (=10 mg/kg). Each bait weighted 2.5 ± 0.1 g. The animals received the baits in their individual feeders. Stainless steel trays were placed beneath each cage to improve visibility in case the animals dropped the bait outside the cage. If the bait was dropped, it was promptly retrieved and returned to the feeder. All steps of bait administration and consumption monitoring were conducted by the same person.

### 2.1. Descriptive Pharmacokinetics (Study I)

Twelve guinea pigs (six males and six females) were included in this study.

On day 0, each animal received one bait containing fluazuron.

On days −7 (pre-treatment) and post-treatment on days 0 + 8 h, +1, +4, +7, +11, +15, +21, and +28, blood samples were collected by jugular venipuncture into heparin tubes.

The samples were centrifuged at 756 g for 10 min at 4 °C, and the plasma obtained was stored at −20 °C until analysis. The pharmacokinetic parameters were determined using a pharmacokinetics solver software (Microsoft Excel^®^, Redmond, WA, USA), applying a non-compartmental model for extravascular administration. The pharmacokinetic parameters were calculated using individual plasma concentration versus time data. The maximum measured concentration for a particular animal (Cmax) and the time from dosing to the maximum concentration (Tmax) were measured individually. The area under the curve from zero to the last time t (AUC0-t) was calculated using the linear trapezoidal method and extrapolated to infinity (AUC0-∞). The results are expressed as the arithmetic mean ± standard deviation (SD). The data were statistically analyzed using one way ANOVA, followed by Tukey’s test for multiple comparisons, with a significance level of 95% (*p* ≤ 0.05) in GraphPad Prism 6.0.

All analytical procedures were performed as described in [10].

### 2.2. Efficacy Against Amblyomma sculptum Larvae (Study II)

The unfed larvae of *A. sculptum* used to infest the guinea pigs were obtained from the colonies maintained in rabbits in the Laboratory for Experimental Chemotherapy in Veterinary Parasitology of Federal Rural University of Rio de Janeiro.

After evaluating the results obtained from Study I, the experimental design of Study II was developed.

Thirty-two guinea pigs (sixteen males and sixteen females) were included in this study.

On day −28 (pre-infestation), the animals were divided into four groups: a control group (CG)—untreated; and three treated groups. One bait was administered to each animal on days −21, −14, and −7 (pre-infestation) to Group 1 (G1), Group 2 (G2), and Group 3 (G3), respectively.

On day −1, a calico bag was attached on the back of each guinea pig. On day 0, all guinea pigs were infested with ±1000 unfed larvae of *A. sculptum* each. The calico bags were inspected daily, and the naturally detached engorged larvae were collected, counted, evaluated, and transferred to an incubator at 27 °C and 85% RH. Fourteen days after incubation, the larvae were evaluated and counted as alive or dead (the infestation and evaluation steps were performed as described by [9]).

To compare the numbers of engorged larvae between the four experimental groups, the Shapiro–Wilk test was initially used to determine the type of data distribution (parametric or nonparametric). It was found that the distribution was nonparametric, so the data were transformed using Log10. After transforming the data, the Shapiro–Wilk test was used again, confirming that the data followed a normal distribution. Then, the one-way ANOVA test was used to compare the averages.

To determine whether there was a significant difference in the efficacy of fluazuron in interrupting of the molt from engorged larvae to nymph, the molt percentages were compared. For this comparison, one-way ANOVA was used. In all analyses, a significance level of ≥95% was considered. Statistical analyses were performed using the Bioestat software [10].

## 3. Results

### 3.1. Descriptive Pharmacokinetics (Study I)

**Figure 1:**
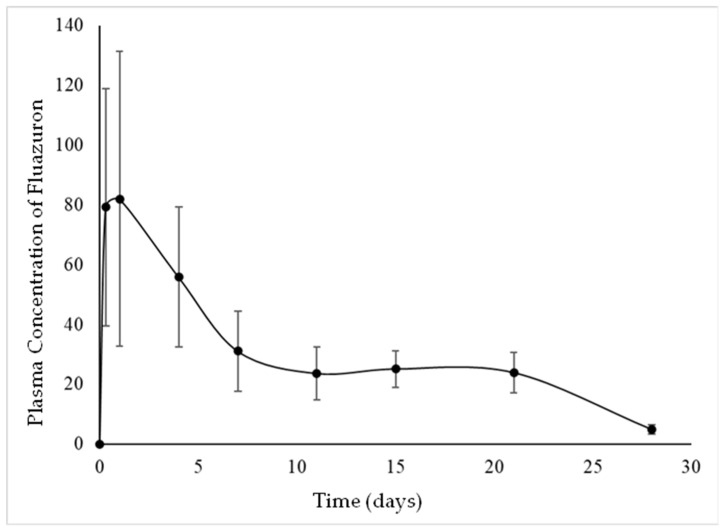
Mean ± SD plasma concentration of fluazuron following orally administration of fluazuron bait (10 mg/kg) to guinea pigs (n = 12).

The mean values and curve of the plasma concentration of fluazuron versus time after single-dose treatment with the bait at a dose of 10 mg/kg of fluazuron are shown in Table 1 and Figure 1, respectively.

Fluazuron plasma concentrations increased rapidly, indicating rapid absorption, reaching Cmax of 107.23 ± 46.69 ng/mL in 0.90 ± 1.04 days (Tmax), with absorption intervals (AUC0-t) of 862.99 ± 182.05, ng/mL*d, and slow elimination with t1/2 of 6.63 ± 2.02 days (Table 2).

### 3.2. Efficacy Against Amblyomma sculptum Larvae (Study II)

The efficacy results are shown in Table 3.

The bait administered containing fluazuron at a dose of 10 mg/kg did not interfere with the detachment of engorged larvae. The average number of engorged larvae among the four experimental groups did not differ significantly (*p* > 0.05).

The observed data indicated efficacy levels of 64.99% (day +7), 61.88% (day +14), and 59.31% (day +21). The statistical analysis demonstrated a statistical difference in the mean values for CG compared with G1, G2, and G3 (*p* ≤ 0.05), while no significant differences were observed among the treated groups (*p* > 0.05).

## 4. Discussion

It is possible to observe that the mean plasma concentrations obtained by [9] and in this study (Study I) follow a similar pattern, with a plasma peak reached within the first 24 h (indicating rapid absorption) followed by slow excretion that can be observed over subsequent days. However, the mean plasma concentrations observed in animals treated with the bait are at least three times lower than in animals treated by gavage in [9], demonstrating that the bait formulation interfered with fluazuron absorption. This may have occurred because the paraffin present in the formulation prevented the release of the drug due to it has water-resistant properties, or because it passed faster than the animals’ gastrointestinal tract can digest it, not allowing enough time for full absorption of the drug.

Since all this work aimed at the future possibility of providing baits containing fluazuron to capybaras, an experimental design was developed for Study II to verify whether the baits could be administered every 7, 14, or 21 days to control the tick *A. sculptum* in guinea pigs. In this study, the animals were not treated on the same day; day zero was set as the day of infestation to minimize the number of animals used.

In the results obtained in Study II, as well as by [9], it was possible to observe that fluazuron administered at a dose of 10 mg/kg, in the form of bait, did not interfere with the recovery of engorged larvae. However, the same high levels of efficacy in interrupting the process of larval molting into nymphs were not observed.

When observing that there was no statistical difference in descriptive pharmacokinetics (Study I) between the mean plasma concentration values for days +7, +15, and +21, it is possible to explain the lack of statistical difference in efficacy between the experimental groups of Study II by noting that, since there was no difference in the mean plasma levels of fluazuron on the different treatment days, the efficacy levels likewise showed no variation.

In a study conducted with rabbits treated topically with different doses of fluazuron [11], the authors observed differences in engorged *Rhipicephalus sanguineus* nymphs between the control and treated groups—differences that were neither observed in this study nor reported by [9]. The differences observed were as follows: longer molting period in the treated groups; smaller engorged specimens recovered in the treated groups; the first engorged nymphs to be released were more affected (killed) by fluazuron in the groups that received doses of 15 mg/kg and above. It is possible that if the guinea pigs had received doses higher than 15 mg/kg, the *A. sculptum* larvae would have been more affected, and larvicidal efficacy could have been observed.

In this study, the main parameter used to evaluate treatment efficacy was the percentage of larval-to-nymphal molt, which is justified by the fact that this is the primary mechanism of action of fluazuron, a chitin synthesis inhibitor. However, in future studies involving other classes of acaricides with distinct mechanisms of action, we recommend additional evaluation of other biological parameters—such as post-engorgement larval weight, feeding rates, and larval mortality, among others—to broaden the understanding of the systemic effects of the substances tested.

From the pharmacokinetic data, it is evident that the bait formulation negatively affected the efficacy of fluazuron. Therefore, increasing the dose may enhance efficacy in interrupting the larval-to-nymphal molt and extend the period of action, possibly exceeding 21 days.

Although *A. sculptum* larvae predominate in the fall and winter [4,12] and their behavior in the environment includes diapause, Study II occurred in the spring without complications, since the larvae used came from a colony maintained under controlled temperature, humidity, and photoperiod conditions.

Fluazuron administered orally to guinea pigs can also be considered successful in controlling *A. sculptum* ticks when the appropriate dose is used, and not only when administered topically to other animals for the control of monoxenous ticks [13,14]. The therapeutic and residual efficacy of fluazuron administered topically was considerably reduced in animals exposed to rain [15]; therefore, fluazuron administered orally has an advantage over topical use when administered to animals that may be exposed to rain or are semi-aquatic (e.g., capybaras [16]).

Fluazuron, like other insect growth regulators, has been widely used orally to control ectoparasites in rodents. However, only the studies by [9,17] directly administered the fluazuron solution orally.

Drug delivery systems have been widely used to control ectoparasites in rodents using baits, but these systems are not always effective. While each bait contained 5 mg of fluazuron and an efficacy of between 59 and 64.99% was obtained in this study, in the study carried out by [8] with rats, each bait contained 40 mg of fluazuron, and while no efficacy was obtained for tick control, it was effective for flea control. The explanation for these divergent results may be that the efficacy was evaluated in different species of rodents and/or ticks.

No adverse reactions were observed in the studies carried out with guinea pigs—all animals remained clinically healthy throughout the experimental period.

The findings of this study allowed us to highlight the potential of fluazuron to control *A. sculptum* through its administration in bait formulation, intended for use in wild rodents in their natural environment. However, future studies should be conducted to find a dosage whose effectiveness exceeds 90%.

Based on the results obtained, guinea pigs represent a suitable experimental model for evaluating the potential of systemically acting molecules in the control of *A. sculptum* in wild rodents as an initial step in the development of oral formulations applicable to wild rodents.

## 5. Conclusions

Pharmacokinetic data demonstrated rapid absorption and slow excretion of fluazuron administered as bait at a dose of 10 mg/kg of body weight, and efficacy data demonstrated potential for use in controlling *A. sculptum* in wild rodents. However, doses higher than 10 mg/kg will need to be evaluated.

## Figures and Tables

**Table 1:** Mean ± SD of plasma concentration of fluazuron (ng·mL^−1^) in guinea pigs treated orally with a single dose of a bait containing fluazuron (10 mg/kg).

Experimental Day	D−7	D0+8 h	D+1	D+4	D+7	D+11	D+15	D+21	D+28
Mean ± SD	0	79.24 ± 39.73 ^a^	81.99 ± 49.30 ^a^	55.86 ± 23.51 ^a^	31.08 ± 13.41 ^b^	23.66 ± 8.81 ^b^	25.15 ± 6.09 ^b^	23.95 ± 6.76 ^b^	4.97 ± 1.58 ^c^

Superscript letters with the same values are not significant (*p* > 0.05); superscript letters with different values are not significant (*p* ≤ 0.05).

**Table 2:** Pharmacokinetics parameters of fluazuron in guinea pigs treated orally with a single dose of a bait containing fluazuron (10 mg/kg) (n = 12).

Pharmacokinetic Parameters	Arithmetic Mean ± SD
C_max_ (ng/mL)	107.23 ± 46.69
T_max_ (d)	0.90 ± 1.04
AUC0-t (ng.d/mL)	862.99 ± 182.05
AUC0-∞ (ng.d/mL)	911.65 ± 181.09
T_1/2_ (d)	6.63 ± 2.02

T_max_—time to reach peak plasma concentration; C_max_—peak plasma concentration; AUC—area under the (zero moment) curve from time 0 to the last detectable concentration; T_1/2_—terminal half-life; AUC—area under the (zero moment) curve from time 0 to infinity.

**Table 3:** Evaluation of detached and molting process of engorged larvae of *Amblyomma sculptum* recovered from artificially infested guinea pigs in the control and the fluazuron-treated groups.

Groups	Average of Engorged Larvae Detached	Larvicidal Efficacy (%)	Average of Molted Nymphs	Molted Nymphs (%)	Molting Process Inhibition (%)
GC	546.6 ± 379.6 ^a^	---	380.0 ± 239.9	70.8 ± 6.6 ^a^	---
G1	790.9 ± 570.8 ^a^	0	254.5 ± 292.3	24.8 ± 13.2 ^b^	64.99
G2	573.1 ± 278.7 ^a^	0	192.9 ± 180.4	27.0 ± 18.7 ^b^	61.88
G3	626.4 ± 297.6 ^a^	0	200.3 ± 113.6	28.8 ± 12.3 ^b^	59.31

The same superscript letters in the same column indicate no significant difference between the arithmetic means (*p* > 0.05); different superscript letters in the same column indicate a significant difference between the arithmetic means (*p* ≤ 0.05). CG—control group (received no treatment); G1—received the bait 21 days before infestation; G2—received the bait 14 days before infestation; G3—received the bait 7 days before infestation.

## Data Availability

The datasets generated or analyzed during the present study are available on request from the corresponding author D.A.B.

## Abbreviations

The following abbreviations are used in this manuscript:

Cmax	Maximum measured concentration
Tmax	Time from dosing to the maximum concentration
AUC0-t	Area under the curve from zero to last time t
AUC0-∞	Area under the curve from zero to infinity
SD	Standard deviation
CG	Control Group
G1	Group 1
G2	Group 2
G3	Group 3

## References

[1] Nava S., Beati L., Labruna M.B., Cáceres A.G., Mangold A.J., Guglielmone A.A. (2014). Reassessment of the taxonomic status of *Amblyomma cajennense* (Fabricius, 1787) with the description of three new species, *Amblyomma tonelliae n*. sp., *Amblyomma interandinum n.* sp. and *Amblyomma patinoi n*. sp., and reinstatement of 15 *Amblyomma mixtum* Koch, 1844, and *Amblyomma sculptum* Berlese, 1888 (Ixodida: Ixodidae). Ticks Tick. Borne Dis..

[2] Szabó M.P.J., Pinter A., Labruna M.B. (2013). Ecology, biology and distribution of spotted-fever tick vectors in Brazil. Front. Cell. Infect. Microbiol..

[3] Labruna M.B., Kasai N., Ferreira F., Faccini J.L.H., Gennari S.M. (2002). Seasonal dynamics of ticks (Acari: Ixodidae) on horses in the state of São Paulo Brazil. Vet. Parasitol..

[4] Ramírez-Hernández A., Uchoa F., de Azevedo Serpa M.C., Binder L.C., Souza C.E., Labruna M.B. (2020). Capybaras (Hydrochoerus hydrochaeris) as amplifying hosts of *Rickettsia rickettsii* to *Amblyomma sculptum* ticks: Evaluation during primary and subsequent exposures to *R. rickettsii* infection. Ticks Tick. Borne Dis..

[5] Ferraz K.M.P., Ferraz S.F.B., Moreira J.R., Couto H.T.Z., Verdade L.M. (2007). Capybara (*Hydrochoerus hydrochaeris*) distribution in agroecosystems: A cross-scale habitat analysis. J. Biogeogr..

[6] Davis R.M. (1999). Use of orally administered chitin inhibitor (lufenuron) to control flea vectors of plague on ground squirrels in California. J. Med. Entomol..

[7] Slowik T.J., Lane R.S., Davis R.M. (2001). Field trial of systemically delivered arthropod development-inhibitor (fluazuron) used to control woodrat fleas (Siphonaptera: Ceratophyllidae) and ticks (Acari: Ixodidae). J. Med. Entomol..

[8] Davis R.M., Cleugh E., Smith R.T., Fritz C.L. (2008). Use of a chitin synthesis inhibitor to control fleas on wild rodents important in the maintenance of plague, Yersinia pestis, in California. J. Vector Ecol..

[9] Borges D.A., Cid Y.P., Magalhães V.D.S., Alves M.C.C., Ferreira T.P., Bonfim I.V., Lima E.A.S., Freitas J.P., Scott F.B. (2022). Fluazuron orally administered to guinea pigs: Pharmacokinetic and efficacy against *Amblyomma sculptum*. Parasit. Vectors.

[10] Ayres M., Ayres J., Ayres D.L., Santos A.S. (2011). BioEstat 5.3: Aplicações Estatísticas nas Áreas das Ciências Biológicas e Médicas.

[11] Oliveira P.R., Calligaris I.B., Roma G.C., Bechara G.H., Pizano M.A., Mathias M.I.C. (2012). Potential of the insect growth regulator, fluazuron, in the control of *Rhipicephalus sanguineus* nymphs (Latreille, 1806) (Acari: Ixodidae): Determination of the LD95 and LD50. Exp. Parasitol..

[12] Oliveira P.R., Borges L.M.F., Lopes C.M.L., Leite R.C. (2000). Population dynamics of the free living stages of *Amblyomma cajennense* (Fabricius, 1787) (Acari: Ixodidae) on pastures of Pedro Leopoldo, Minas Gerais State, Brazil. Vet. Parasitol..

[13] Graf J.F. (1993). The role of insect growth regulators in arthropod control. Parasitol. Today.

[14] Junquera P., Hosking B., Gameiro M., Macdolnal A. (2019). Benzoylphenyl ureas as veterinary antiparasitics. An overview and outlook with emphasis on efficacy, usage and resistance. Parasite.

[15] Zapa D.M.B., Couto L.F.M., Heller L.M., De Assis Cavalcante A.S., Nicaretta J.E., Cruvinel L.B., Melo Júnior R.D., Ferreira L.L., Bastos T.S.A., Soares V.E. (2020). Do rainfall and tick burden affect the efficacy of pour-on formulations against *Rhipicephalus (Boophilus) microplus*?. Prev. Vet. Med..

[16] Macdonald D.W. (1981). Dwindling resources and the social behaviour of capybaras, (*Hydrochoerus hydrochaeris*) (Mammalia). J. Zool..

[17] Pasay C., Rothwell J., Mounsey K., Kelly A., Hutchinson B., Miezler A., McCarthy J. (2012). An exploratory study to assess the activity of the acarine growth inhibitor, fluazuron, against *Sarcoptes scabei* infestation in pigs. Parasit. Vectors.

